# Rice resistance to lepidopteran herbivores is enhanced by overexpression of a key transcription factor gene for diterpenoid phytoalexin biosynthesis

**DOI:** 10.5511/plantbiotechnology.25.1023a

**Published:** 2026-03-25

**Authors:** Yasukazu Kanda, Kazumu Kuramitsu, Akira Takahashi, Mai Tsuda, Yooichi Kainoh, Masaki Mori

**Affiliations:** 1 Institute of Agrobiological Sciences, National Agriculture and Food Research Organization (NARO), Tsukuba, Ibaraki 305-8634, Japan; 2 Department of Frontier Research and Development, Kazusa DNA Research Institute, Kisarazu, Chiba 292-0818, Japan; 3 Faculty of Life and Environmental Sciences, University of Tsukuba, Tsukuba, Ibaraki 305-8572, Japan; 4 Tsukuba-Plant Innovation Research Center (T-PIRC), University of Tsukuba, Tsukuba, Ibaraki 305-8572, Japan

**Keywords:** chewing herbivores, diterpenoid phytoalexin, fall armyworm, pest resistance, rice

## Abstract

In the midst of a worldwide outbreak of lepidopteran chewing pests such as the fall armyworm (FAW; *Spodoptera frugiperda*), innovative strategies for pest control are needed. Recent findings have linked the resistance of rice (*Oryza sativa*) to lepidopteran pests to diterpenoid phytoalexins (DPs), antimicrobial compounds produced in response to disease and abiotic stress conditions. In this study, we explored the involvement of DP biosynthesis-related genes in the response and resistance of rice plants to lepidopteran pests. In interactions between rice and FAW or the oriental armyworm (*Mythimna separata*), feeding damage from larvae induced the expression of *DITERPENOID PHYTOALEXIN FACTOR* (*DPF*), which encodes a key transcription factor in DP biosynthesis, as well as DP biosynthetic genes. Transcriptional analysis suggested that DPF promotes DP biosynthesis in response to chewing by lepidopteran herbivores and other stresses. When lepidopteran larvae were reared on the leaves of *DPF*-overexpressing transgenic rice plants, which persistently accumulate high DP levels, FAW larvae exhibited poor growth within days. Overexpression of *DPF* also suppressed the growth of larvae from the oriental armyworm, although the suppression was more moderate. These results demonstrate that *DPF* overexpression enhances plant resistance to lepidopteran pests, highlighting the potential of *DPF* as a tool for biotechnological pest control.

## Introduction

Arthropod herbivores threaten food production worldwide by feeding on crops (Oerke 2006). They feed on plants through chewing (e.g., caterpillars and grasshoppers) or via piercing and sucking (e.g., aphids). The larvae of lepidopteran insects (moths and butterflies) can be severe chewing pests. For example, the oriental armyworm (OAW; *Mythimna separata* [Walker]) is a chewing pest distributed in Asia and Australasia (Hirai 1988). This species occurs in sudden outbreaks and severely damages rice (*Oryza sativa*), maize (*Zea mays*), and other crops (Hirai 1988). Another lepidopteran insect, the fall armyworm (FAW; *Spodoptera frugiperda* [J.E. Smith]), is a major invasive pest. The species is native to the Americas and has rapidly spread worldwide, including Africa, Asia, and Australia since 2016 (Overton et al. 2021; Stokstad 2017). FAW was first detected in China in early 2019 and in Japan in July 2019 (Wu et al. 2022). The larvae of FAW feed on a wide range of crops, including maize, sorghum (*Sorghum bicolor*), and rice, causing enormous economic losses (Overton et al. 2021). In 12 African maize-producing countries alone, these losses are estimated to affect 21–53% of annual maize production or US$2.5–6.3 billion per year (Day et al. 2017). With substantial recent pest outbreaks, pest control is becoming increasingly important. For many years, synthetic insecticides have been used to control pests (Oerke 2006). Transgenic crops, such as insect-resistant *Bt* crops that express *Bacillus thuringiensis* toxins, also have been widely commercialized (Xiao and Wu 2019). However, arthropod pests acquire tolerance to insecticides and proteins conferring resistance to insects (Hafeez et al. 2022; Xiao and Wu 2019), underscoring the need for the development of new pest control methods.

Plants naturally produce secondary metabolites that enable them to adjust to stressful environments. In rice, disease recognition triggers the biosynthesis and accumulation of a group of antimicrobial low-molecular-weight compounds named diterpenoid phytoalexins (DPs) (Schmelz et al. 2014). Various DPs are produced in rice plants through a biosynthetic pathway including enzymes such as *ENT*-COPALYL DIPHOSPHATE SYNTHASE (CPS2), *SYN*-COPALYL DIPHOSPHATE SYNTHASE (CPS4), and KAURENE-SYNTHASE LIKE (KSL) (Schmelz et al. 2014). The key transcription factor DITERPENOID PHYTOALEXIN FACTOR (DPF; also reported as basic helix-loop-helix 25 [bHLH25] and encoded by Os01g0196300) induces the transcription of DP biosynthetic genes (Yamamura et al. 2015). The transcriptional activation of DP biosynthetic genes and accumulation of DP, which are induced by rice blast or abiotic stresses, are largely dependent on *DPF* (Ishikawa et al. 2024). Importantly, overexpression of *DPF* causes the constitutive accumulation of DP in rice plants (Yamamura et al. 2015). Accumulated DPs serve as a chemical defense against microorganisms. For instance, knockout of *OsCPS2* raised the susceptibility to the rice blast fungus *Pyricularia oryzae* and to the rice blight bacterium *Xanthomonas oryzae* pv. *oryzae* (Lu et al. 2018; Zhang et al. 2021). Rice plants overexpressing *OsCPS2* or *OsCPS4* exhibited enhanced resistance to rice blight (Lu et al. 2018; Zhang et al. 2021). Knockout of *DPF* also lowered the resistance of rice plants to rice blast, whereas its overexpression enhanced it (Liao et al. 2025). *dpf* knockout rice plants were also more susceptible to the parasitic nematode *Meloidogyne graminicola* (Desmedt et al. 2022).

DPs in rice have recently been associated with resistance to lepidopteran chewing herbivores. Overexpression of the gene encoding the signaling protein BROAD-SPECTRUM RESISTANCE 1 (BSR1) promoted the accumulation of DPs in rice following feeding by the lepidopteran herbivore *Mythimna loreyi* (Duponchel), which slightly increased plant resistance to the pest (Kanda et al. 2019, 2023; Sugano et al. 2018). Feeding on an artificial diet containing momilactone B, a rice DP, suppressed the growth of *M. loreyi* larvae, suggesting that rice DPs contribute to the defense mechanism against chewing herbivores (Kanda et al. 2023). These findings suggest that DP biosynthesis-related factors may serve as a tool to control pests, which we tested in this study by challenging rice plants overexpressing *DPF* with feeding by OAW and FAW larvae.

## Materials and methods

### Plant materials

Rice (*Oryza sativa* ssp. *japonica* ‘Nipponbare’) was used as the wild type (WT) in all experiments. The two knockout lines of *DPF* used in this study were previously reported (Ishikawa et al. 2024). For *DPF*-overexpressing (*DPF*-OX) lines, a previously reported line (OX1) (Yamamura et al. 2015) and another line (OX6), which was generated according to the same method as OX1, were used. Briefly, these lines overexpress the full-length coding sequence of *DPF* (AK102964) under the control of the maize *Ubiquitin* promoter. Rice plants were grown under a 16 h light/8 h dark photoperiod at 25°C in a plant cultivation room.

### Insect collecting and rearing

The original FAW colony was collected from maize and sorghum fields in Kagoshima Prefecture, Kyushu, Japan, between July 11 and July 16, 2023. The original OAW colony was obtained from stock cultures maintained in the Laboratory of Applied Entomology and Zoology, University of Tsukuba, Japan. Both FAW and OAW colonies were reared on an artificial diet (Silkmate® 2M; Nosan Corporation, Yokohama, Japan) under laboratory conditions (25±1°C, 16 h light/8 h dark photoperiod, and 60±10% relative humidity). Second-instar larvae (5–7 mg each) were used for all experiments.

### Inoculation with larvae and larva performance assays

For the inoculation, FAW and OAW larvae were transferred to an empty Petri dish and starved for about 10 h, after which larvae of similar weight were collected. Rice plants at 10th–13th of leaf stage were used for inoculation. Intact similar-sized leaves were harvested from rice plants at the same leaf stage and the cut ends were covered with wet KimWipes. Each leaf was placed in a Petri dish and inoculated either with a larva (“cut+feeding” sample) or without a larva (“cut” sample, as a mock treatment) and incubated under a 16 h light/8 h dark photoperiod at 25°C. Larvae were weighed at 2 and 4 days after inoculation for larval performance assays.

### Reverse transcription quantitative PCR (RT-qPCR)

Leaves from rice plants were inoculated with or without larvae as described above and then frozen in liquid nitrogen at 24 h after the beginning of incubation. To prepare non-treated samples, intact leaves were harvested and immediately frozen in liquid nitrogen. Total RNA was extracted from the frozen leaves using Sepasol-RNA I Super G (Nacalai Tesque, Kyoto, Japan) and reverse transcription quantitative PCR (RT-qPCR) was performed as previously described (Kanda et al. 2019). Relative transcript levels of target genes were calculated using the comparative C_T_ (2^−ΔΔCt^) method with rice *UBIQUITIN1* (*RUBQ1*) as the reference gene (Jiang et al. 2010; Livak and Schmittgen 2001). Primers used in this study are listed in Supplementary Table S1.

### Statistical analysis

One-way analysis of variance (ANOVA) followed by Tukey’s honestly significant difference (HSD) test was performed in R (https://www.R-project.org/ (Accessed Apr 4, 2025) using RStudio 2024.12.1+563 for Windows (https://posit.co/download/rstudio-desktop/ (Accessed Apr 4, 2025). *t*-test was performed using Statcel-the Useful Addin Forms on Excel-4th ed. (OMS, Tokyo, Japan).

## Results

### Larval feeding induces the expression of DP biosynthesis-related genes in the leaves of rice plants

**Figure 1:**
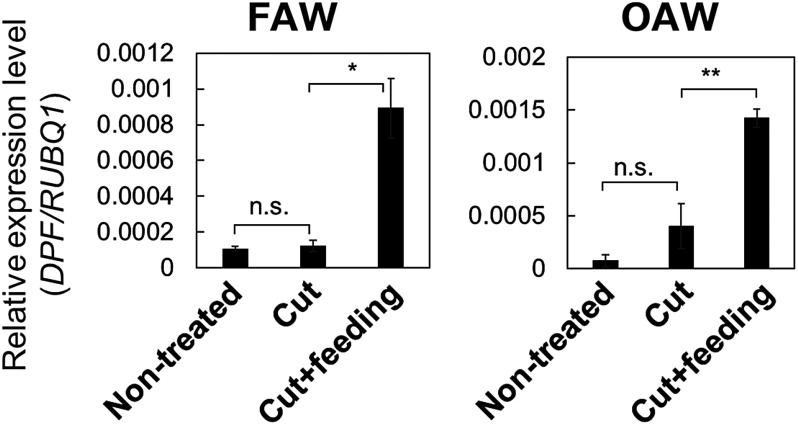
Figure 1. *DPF* expression is induced in response to feeding by FAW and OAW larvae. Rice leaves on which larvae were placed for feeding were sampled 24 h later. Values are presented as means±standard deviation (SD) from three technical replicates of one representative experiment. The experiment was conducted twice, and similar results were obtained. Asterisks indicate significant differences between the indicated samples (Welch’s *t*-test; *, *p*<0.05; **, *p*<0.01). n.s., not significant.

**Figure 2:**
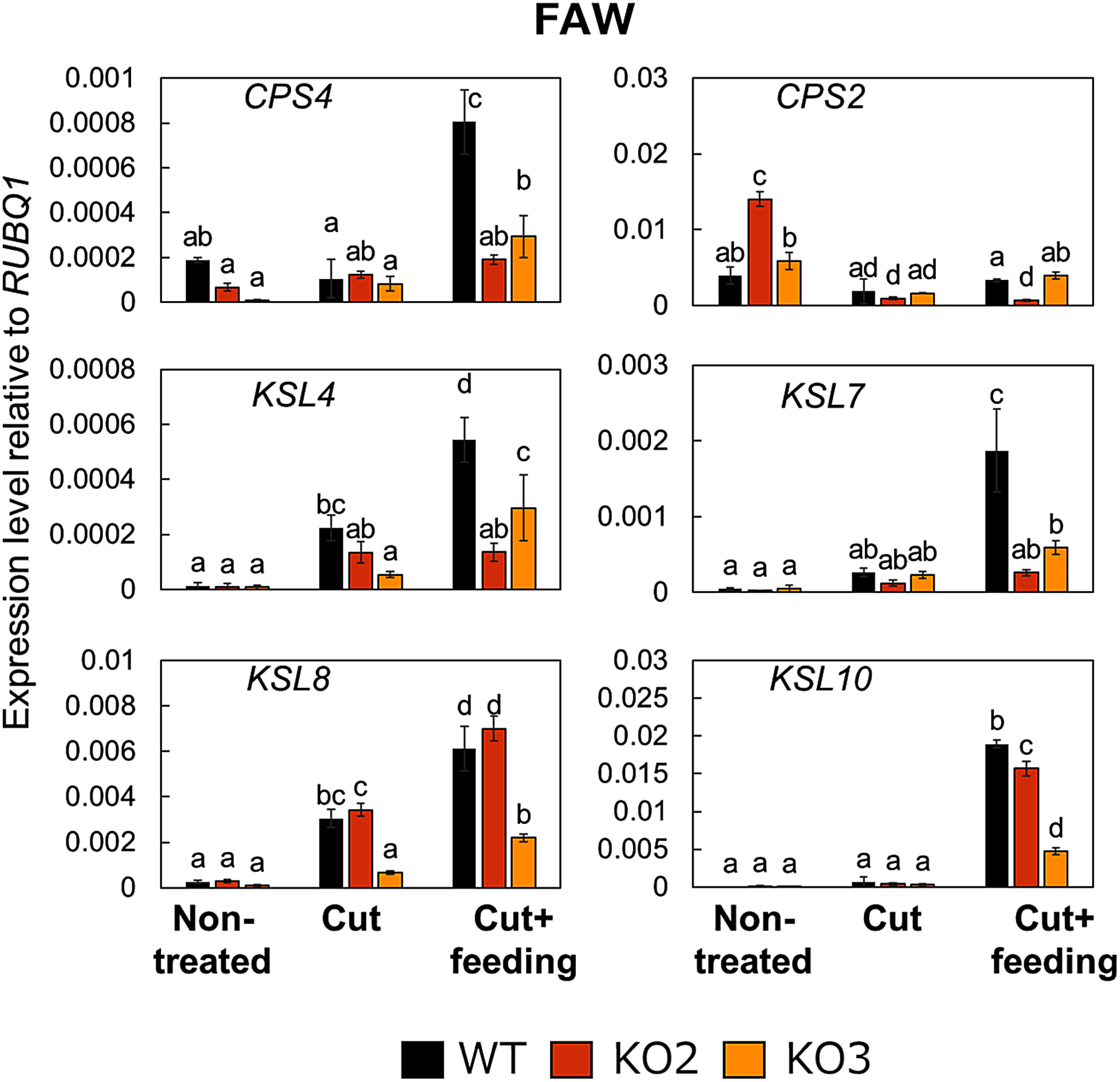
Figure 2. The expression of several DP biosynthetic genes is activated in response to FAW feeding in a largely *DPF*-dependent manner. Rice leaves on which larvae were placed for feeding were sampled 24 h later. KO2 and KO3 are independent *dpf*-knockout lines. Values are presented as means±SD from three technical replicates of one representative experiment. The experiment was conducted twice, and similar results were obtained. Different letters indicate significant differences (*p*<0.05) as determined by a one-way analysis of variance (ANOVA) followed by Tukey’s honestly significant difference (HSD) test. WT, wild type (Nipponbare).

**Figure 3:**
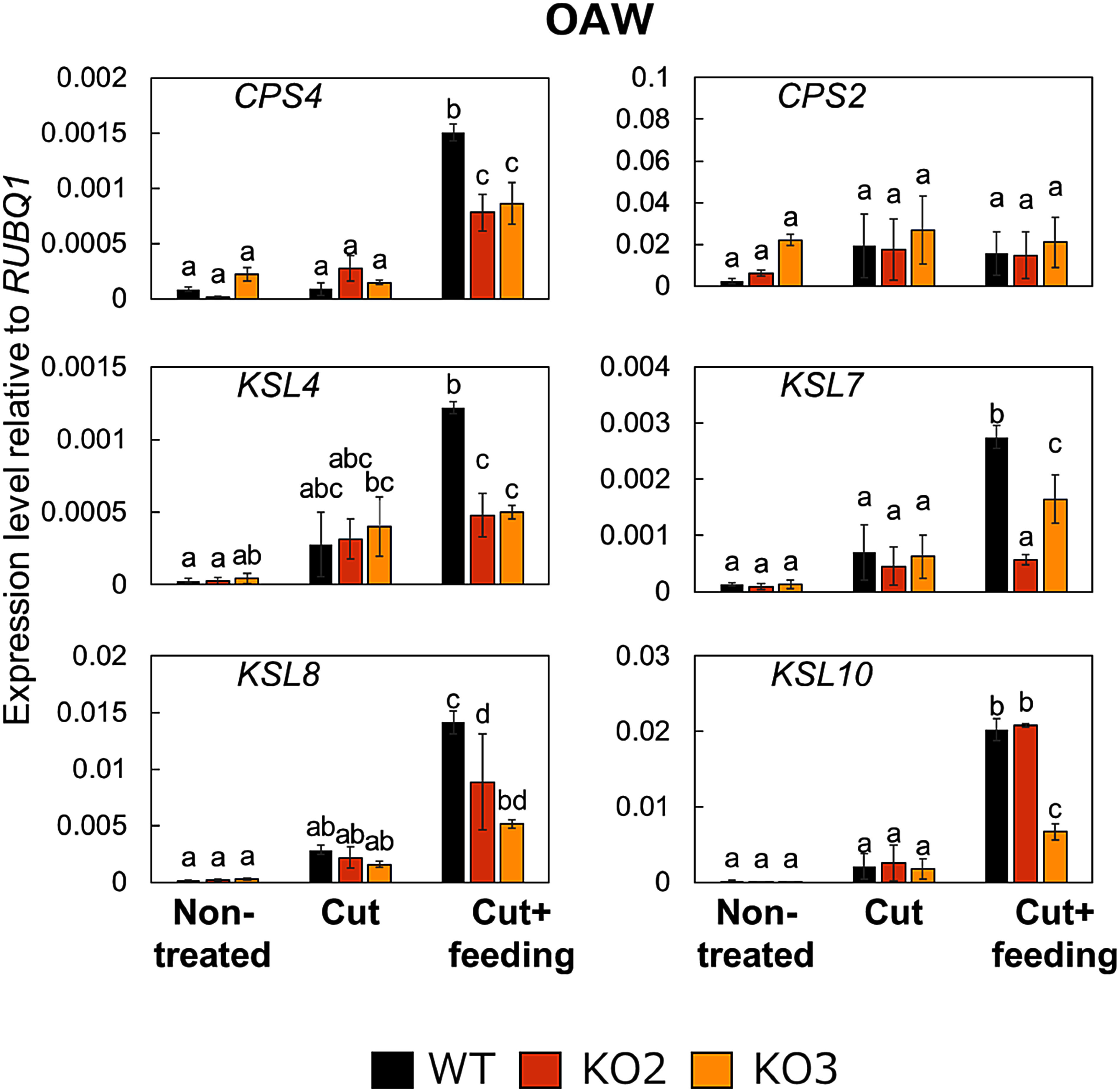
Figure 3. Knocking out *DPF* suppresses the transcript accumulation of DP biosynthetic genes in response to OAW. Rice leaves on which larvae were placed for feeding were sampled 24 h later. KO2 and KO3 are independent *dpf*-knockout lines. Values are presented as means±SD from three technical replicates of one representative experiment. The experiment was conducted twice, and similar results were obtained. Different letters indicate significant differences (*p*<0.05), as determined by one-way ANOVA followed by Tukey’s HSD test. WT, wild type (Nipponbare).

To determine whether the expression of DP biosynthesis-related genes is naturally responsive to chewing herbivores, we measured the expression levels of these genes in rice leaves damaged by feeding from the larvae of lepidopteran insects. To this end, we placed leaves of WT and *dpf*-knockout plants in a Petri dish with a larva (“cut+feeding”) or without a larva (“cut”, used as mock control) before inoculating with FAW or OAW larvae. After 24 h of incubation under a 16 h light/8 h dark photoperiod at 25°C, we collected all inoculated leaves for RT-qPCR analysis, alongside freshly cut leaves from plants not exposed to larvae as an additional control (“non-treated”). In the leaves of WT plants, relative *DPF* transcript levels were similar between mock-treated and non-treated plants, but rose significantly following feeding by FAW or OAW larvae (Figure 1). The DP biosynthetic genes *CPS4*, *KSL4*, *KSL7*, *KSL8*, and *KSL10* also showed greater transcript levels in leaves on which larvae fed than in non-treated or mock-treated leaves, although we observed no change in *CPS2* transcript levels at this time point (Figures 2, 3). These results indicate that the expression of *DPF* and the DP biosynthetic genes is induced during a natural interaction between larvae and rice plants.

We used two knockout lines of *DPF* harboring a 1 bp-insertion in different sites of the *DPF* coding region (Ishikawa et al. 2024) to assess the contribution of DPF to the observed changes in gene expression in response to larval feeding. In the leaves of these *dpf* knockout lines, the transcript levels of *CPS4*, *KSL4*, and *KSL7* were significantly lower than those in the leaves of WT plants in response to feeding by FAW (Figure 2). We obtained similar results following feeding by OAW larvae (Figure 3). Importantly, *CPS4* and *KSL4* encode the representative enzymes for the biosynthetic pathway of momilactone B, which inhibits the growth of lepidopteran larvae (Kanda et al. 2023; Yamane 2013). These results suggest that DPF contributes to the transcriptional activation of DP biosynthetic genes in response to larval feeding, in addition to its previously demonstrated role upon pathogen challenge, consistent with previous studies showing that herbivore-associated molecular patterns (HAMPs) elicit *DPF* expression in cultured rice cells (Kanda et al. 2023). Notably, the knockout of *DPF* did not completely abolish the observed induction of transcript levels for DP biosynthetic genes, with small but statistically significantly higher levels occasionally observed relative to WT (e.g., *CPS4* in Figure 3). Relative *KSL8* and *KSL10* transcript levels were also not always significantly lower in the two knockout lines relative to WT (Figures 2, 3), suggesting the presence of redundant regulation for the activation of DP biosynthetic genes other than via DPF.

### *DPF* overexpression enhances resistance to lepidopteran larvae

**Figure 4:**
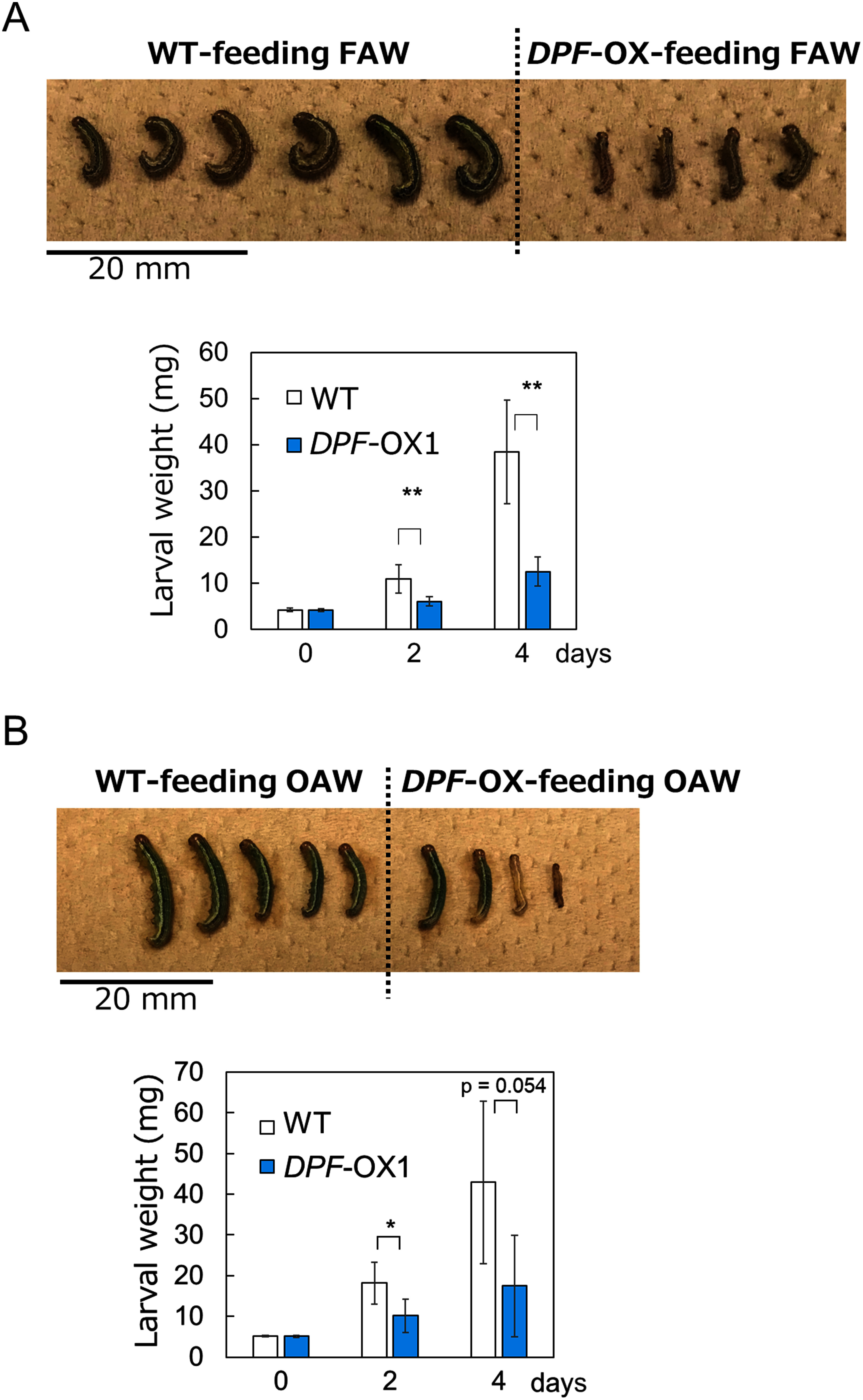
Figure 4. Larvae of FAW and OAW feeding on the leaves of *DPF*-overexpressing plants exhibit poor growth. FAW (A) or OAW (B) larvae reared on the leaves of a *DPF*-overexpressing (OX) rice line exhibit poor growth. Top panels, representative photographs taken after 4 days of rearing; lower panels, average weight of the larvae. Values are presented as means±SD from biological replicates (*n*=4–6) of one representative experiment. The experiment was conducted twice, and similar results were obtained. Asterisks indicate significant differences between the indicated samples (Welch’s *t*-test; *, *p*<0.05; **, *p*<0.01). WT, wild type (Nipponbare).

The observation that the transcript levels of *DPF* and DP biosynthetic genes responded to larval feeding (Figures 1–3) raised the possibility that *DPF* overexpression may enhance resistance to pests. To investigate the possible effect of *DPF* overexpression on pest resistance, we prepared two rice lines overexpressing *DPF* (*DPF*-OX1 and *DPF*-OX6) under the control of the maize *Ubiquitin* (*Ubi*) promoter in the Nipponbare background. *DPF* overexpression was previously shown to lead to the constitutive expression of DP biosynthetic genes, resulting in DP accumulation (Yamamura et al. 2015). We confirmed that *DPF* was expressed at higher levels in the two overexpression lines (Supplementary Figures S1, S2). In a larval inoculation assay with FAW or OAW larvae, RT-qPCR analysis indicated that the DP biosynthetic genes are also expressed at higher levels in the *DPF*-overexpression lines than in the WT (Supplementary Figures S1, S2). When feeding on WT leaves, the weight of FAW larvae increased by 3.0 to 9.1 times over 4 days. By contrast, FAW larvae fed on leaves from the *DPF*-OX1 and *DPF*-OX6 lines showed significantly suppressed growth (Figure 4A, Supplementary Figure S3). The biomass of larvae fed on *DPF*-OX leaves only reached 33–66% of that of larvae fed on WT leaves, demonstrating that *DPF* overexpression enhances resistance to FAW. When we tested the effect of *DPF* overexpression on the feeding of OAW larvae, we observed significant growth inhibition in the *DPF*-OX1 line (Figure 4B, Supplementary Figure S4A), but not in the *DPF*-OX6 line (Supplementary Figure S4B). This difference between the lines may be explained by the lower *DPF* expression levels in *DPF*-OX6 than in *DPF*-OX1 in response to feeding damage (Supplementary Figures S1, S2). Overexpression of *DPF* thus appeared to confer only moderate resistance to OAW, possibly because OAW larvae are more tolerant to rice anti-herbivore compounds, such as momilactone B, than FAW. Taken together, overexpression of *DPF* enhances the resistance to the lepidopteran pests FAW, and OAW to a lesser extent, in rice.

## Discussion

**Figure 5:**
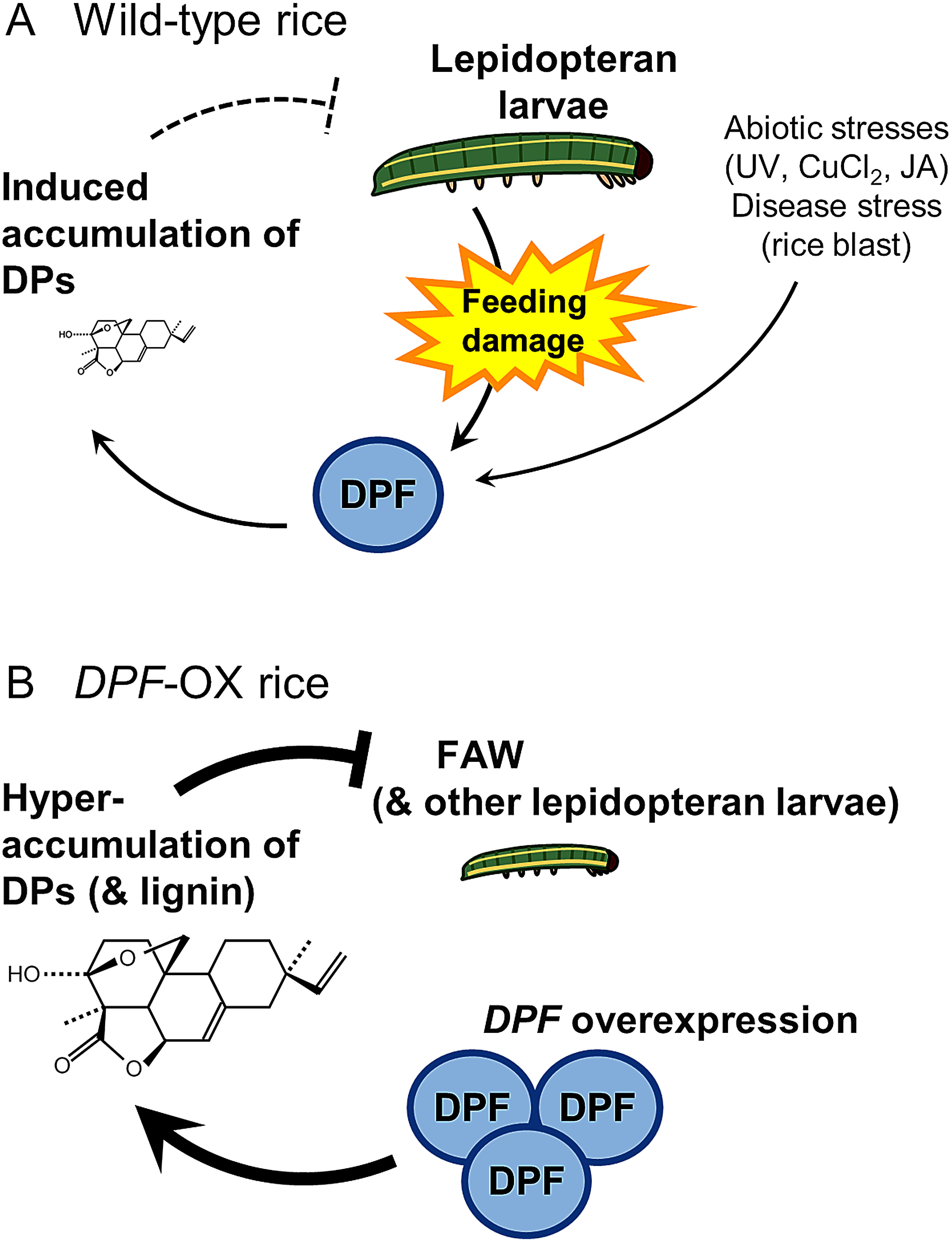
Figure 5. Proposed model of resistance to lepidopteran pests via *DPF* in rice. (A) Diagram showing the proposed mechanism underlying natural resistance to lepidopteran pests by feeding-triggered DPs production. (B) Resistance to FAW and other lepidopteran larvae conferred by *DPF* overexpression. DPs, diterpenoid phytoalexins; JA, jasmonic acid; FAW, fall armyworm.

Previous studies have suggested a positive correlation between the accumulation of DPs in rice and responses triggered by chewing pests; however, studies exploring the effect of caterpillar feeding on DP biosynthetic genes are scarce (Kanda et al. 2023; Schmelz et al. 2014; Yamane 2013). Our results revealed that the expression of DP biosynthetic genes and the transcription factor gene *DPF* respond to chewing by herbivorous insects FAW and OAW (Figures 1–3). Previous study has detected momilactone A and momilactone B in rice plants damaged by the lepidopteran pest *M. loreyi* larvae, consistent with our expression data (Alamgir et al. 2016). Among the analyzed DP biosynthesis genes, the expression level of *CPS2* did not increase under cut+feeding treatment, although it increases in response to infection of the rice blast fungus (Ishikawa et al. 2024). Notably, while CPS2 enzyme consumes geranylgeranyl diphosphate (GGDP) as a substrate to initiate biosynthesis toward phytocassanes and oryzalexins A–F, CPS4 serves as the starting point for the biosynthetic pathway from GGDP to momilactones, including momilactone B, which shows direct growth-inhibitory effect to larvae (Kanda et al. 2023; Schmelz et al. 2014; Yamane 2013). Combined with these findings, our data raise the possibility that rice plants express different DP biosynthetic genes depending on the type of biotic stresses. Alternatively, since this study did not include time-course analysis for expressions, it cannot be ruled out that the timing of *CPS2* expression differs from that of other biosynthetic genes. Further analysis revealed that DPF regulates the expression of DP biosynthetic genes in response to challenges posed by the lepidopteran pests, FAW and OAW (Figures 2, 3). These findings support a model in which rice plants may activate the production of DPs via DPF upon larval feeding, after which the accumulated DPs enhance resistance to herbivores (Figure 5A). The direct inhibition of larval growth by momilactone B, a type of DP, in previous reports (Kanda et al. 2023) is consistent with this model. The quantification of DPs and assessment of resistance to these pests in plants with knockout of DP biosynthesis-related genes will be required to fully support this model.

Importantly for biotechnological applications, overexpression of *DPF* in rice enhanced plant resistance to two severe lepidopteran pests, FAW and OAW (Figure 4). The most plausible cause of the enhanced resistance is the high accumulation of DPs. *DPF*-OX rice plants accumulate high levels of DPs (Yamamura et al. 2015), which may thus contribute to the inhibition of larval growth (Figure 5B). The mechanism of action of DPs on animals remains unknown, except for the anticancer activity of momilactone B in human cultured cells. The anticancer activity is mediated by arresting the cell cycle at the G1 phase and inducing apoptotic cell death (Park et al. 2014). Lepidopteran larvae, which typically grow rapidly, may be more sensitive to the cell cycle-arresting activity than other animals, although it is unclear whether DPs exhibit similar activity in insect cells. Another factor that may contribute to the enhancement of pest resistance is lignin, a cell wall component. In a recent report, rice plants overexpressing *DPF* accumulated higher contents of lignin in addition to DPs when blast fungus infected, resulting in resistance to blast disease (Liao et al. 2025). The mechanical traits of leaves, such as strength and toughness, can function as a defense against herbivory by chewing pests (Caldwell et al. 2016). Reinforcement of the cell wall by lignin deposition may also have contributed to the greater resistance to FAW and OAW observed in *DPF*-OX rice plants.

In the *DPF*-OX lines used in this study, we observed some lesion-mimic brown spots but no marked dwarfism, whereas excessive levels of *DPF* overexpression resulted in severe browning and dwarfism in a previous study (Yamamura et al. 2015). No dwarfism was observed in independent *DPF*-OX rice lines (Liao et al. 2025), indicating that finely tuned *DPF*-OX rice should exhibits the pest resistance and no undesirable phenotypes. In the previous study on the disease resistance gene *BSR1*, undesirable traits caused by *BSR1* overexpression under maize *Ubiquitin* promoter, which is used in this study, were overcome by using different promoters such as moderately expressed *OsUbi7* promoter (Maeda et al. 2024). The same strategy may be applicable when developing *DPF*-overexpressing transgenic varieties in the future. Furthermore, no toxicity or effects of DPs have been reported on humans, except for an antitumor activity for momilactone B (Kim et al. 2007). DPs are organic compounds naturally produced by rice plants (Schmelz et al. 2014; Yamane 2013). Together, these findings support the potential use of finely tuned *DPF*-overexpression lines as transgenic crops resistant to lepidopteran pests. This study sheds light on *DPF* as an innovative tool to prevent the emerging threat of pests.

## Accession numbers

*DPF* (Os01g0196300), *KSL8* (Os11g0474800), *KSL10* (Os12g0491800), *CPS2* (Os02g0571100), *KSL7* (Os02g0570400), *CPS4* (Os04g0178300), *KSL4* (Os04g0179700), and *RUBQ1* (Os06g0681400).

## Description of supplementary files

Supplementary Figure S1. Relative transcript levels of *DPF* and DP biosynthetic genes in *DPF-*overexpressing rice plants in response to treatment with FAW.

Supplementary Figure S2. Relative transcript levels of *DPF* and DP biosynthetic genes in *DPF-*overexpressing rice plants in response to treatment with OAW.

Supplementary Figure S3. *DPF*-overexpressing line OX6 also exhibits greater resistance to FAW.

Supplementary Figure S4. Two *DPF*-overexpressing lines show distinct responses to OAW treatment.

Supplementary Table S1. List of primers used in this study.
