# Supplementary material for: Rice resistance to lepidopteran herbivores is enhanced by overexpression of a key transcription factor gene for diterpenoid phytoalexin biosynthesis
Source: Plant Biotechnol (Tokyo). 2026 Mar 25;43(1):53–9. doi: 10.5511/plantbiotechnology.25.1023a (PMC13170806; doi:10.5511/plantbiotechnology.25.1023a)
Supplement: Supplementary Data [file plantbiotechnology-43-1-25.1023a-s001.pdf]

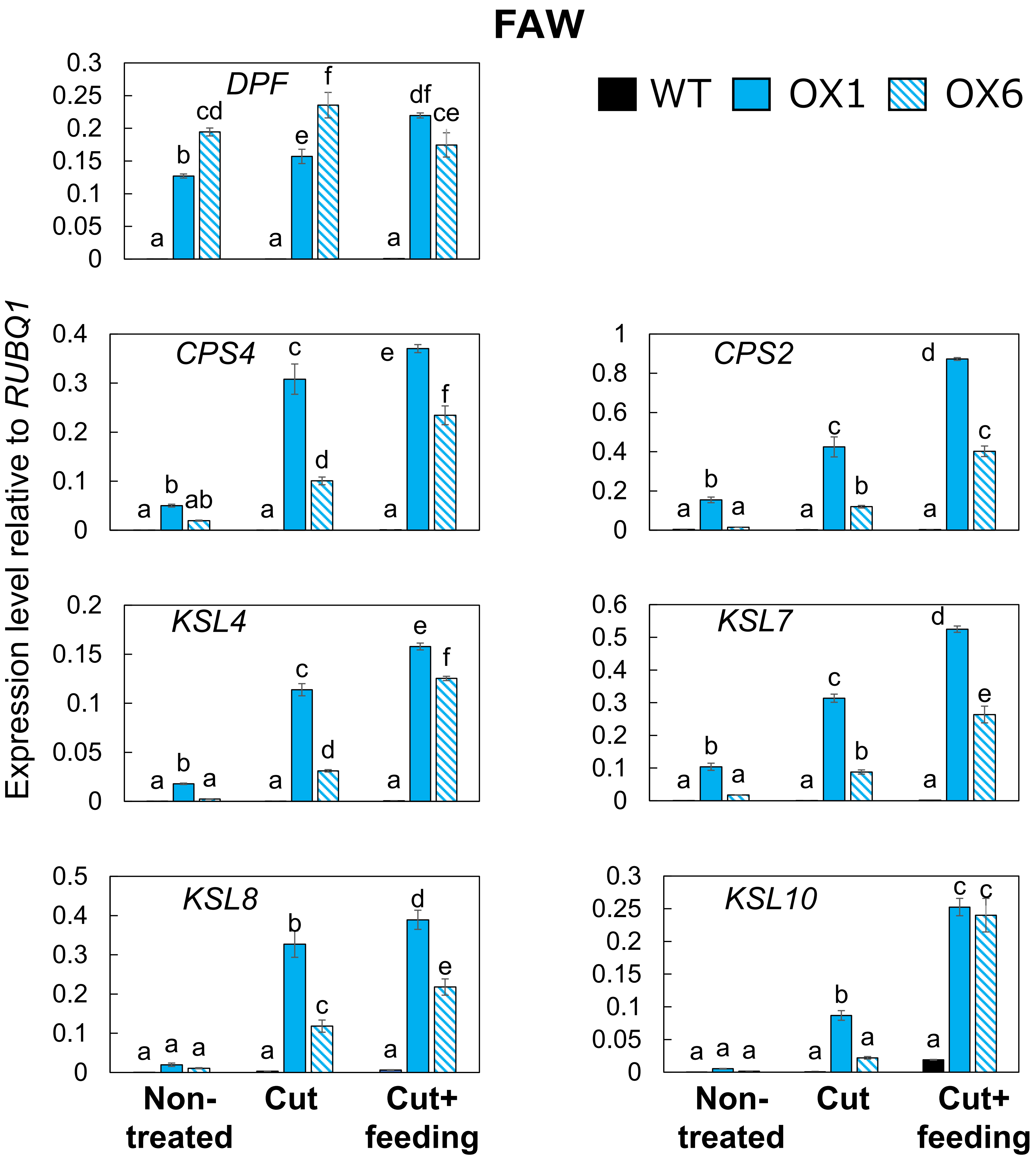

Supplementary Figure S1. Relative transcript levels of *DPF* and DP biosynthetic genes in *DPF*-overexpressing rice plants in response to treatment with FAW. OX1 and OX6 are independent *DPF*-overexpressing lines. Values are presented as the mean ± standard deviation of three biological replicates in one representative experiment. The experiments were conducted twice, and similar results were obtained. Different letters indicate significant differences ($p < 0.05$) calculated using one-way ANOVA followed by Tukey's HSD test. WT, wild type (Nipponbare) rice.

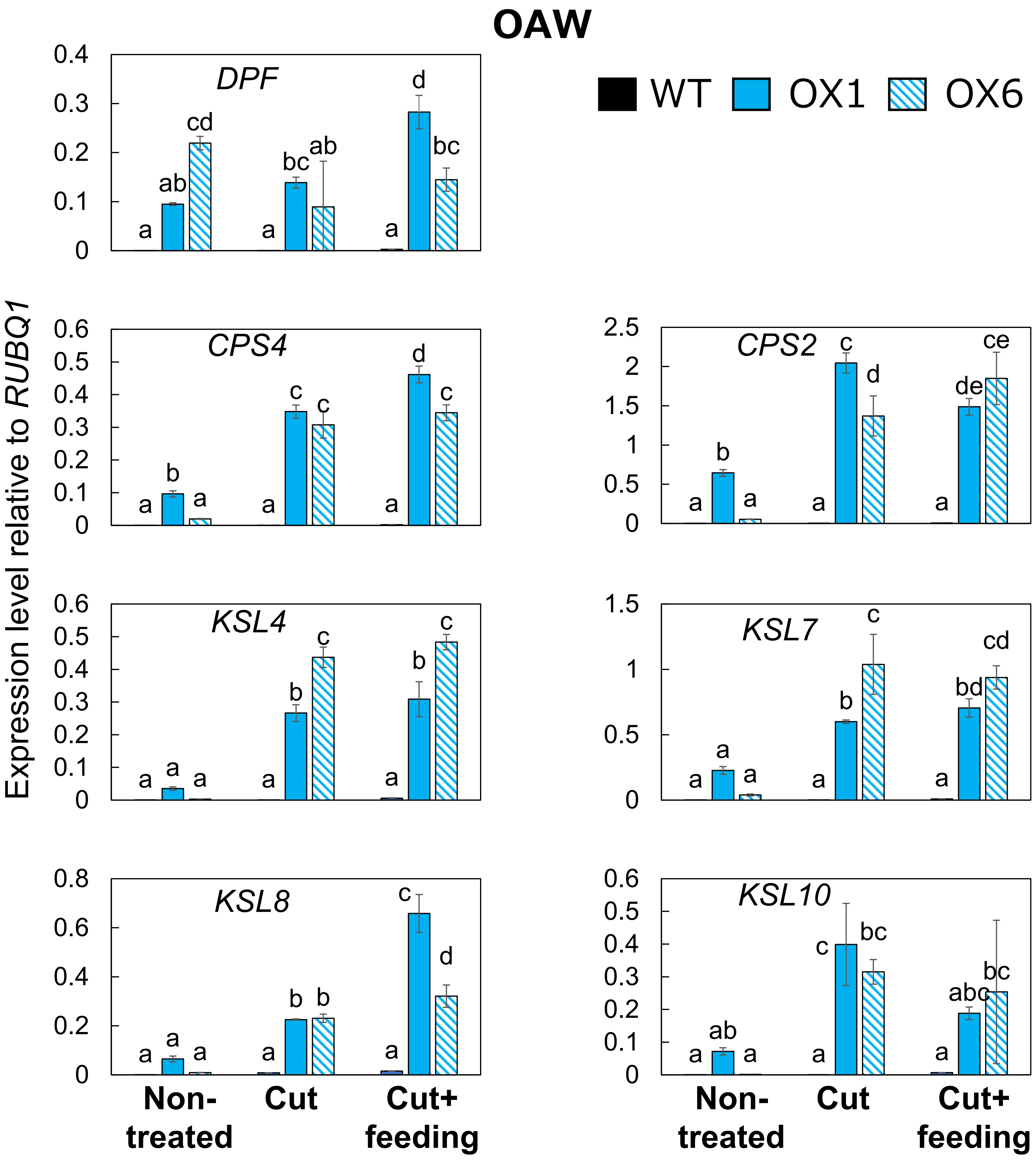

Supplementary Figure S2. Relative transcript levels of *DPF* and DP biosynthetic genes in *DPF*-overexpressing rice plants in response to treatment with OAW. OX1 and OX6 are independent *DPF*-overexpressing lines. Values are presented as the mean ± standard deviation of three biological replicates in one representative experiment. The experiments were conducted twice, and similar results were obtained. Different letters indicate significant differences ($p < 0.05$) calculated using one-way ANOVA followed by Tukey's HSD test.

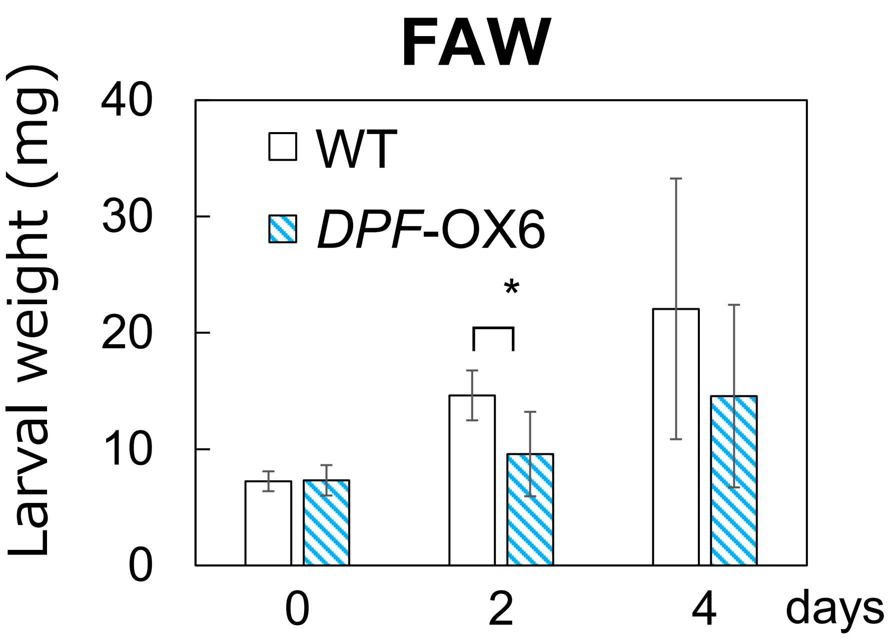

Supplementary Figure S3. *DPF*-overexpressing line OX6 also exhibits greater resistance to FAW. OX6 line, which is the other *DPF*-overexpressing line than OX1 in Figure 1, was used. Values are presented as the mean ± standard deviation of biological replicates (n = 5) in one representative experiment. Asterisks indicate significant differences between the indicated values (Welch's t-test; * $p < 0.05$).

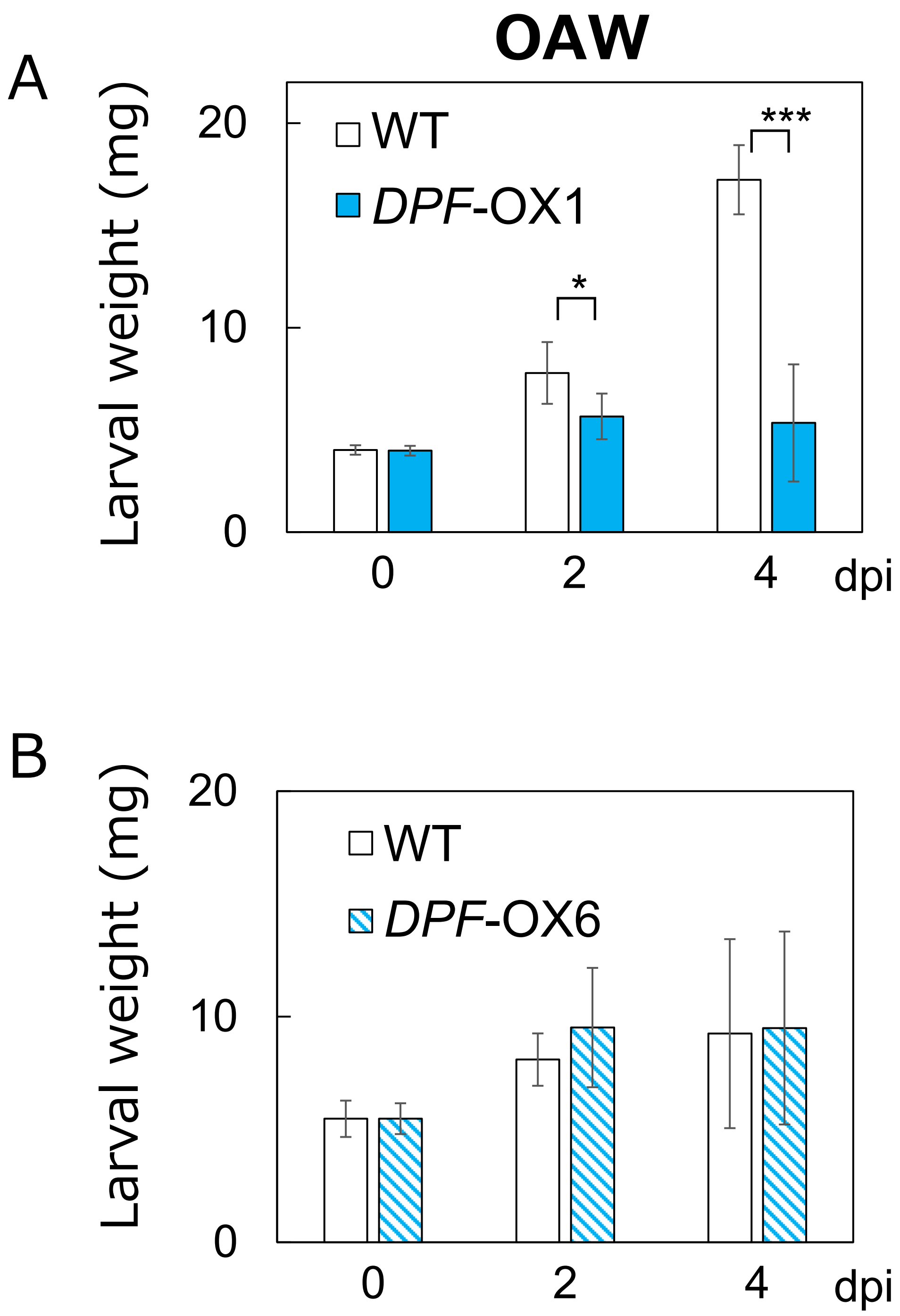

Supplementary Figure S4. Two *DPF*-overexpressing lines show distinct responses to OAW treatment. The *DPF*-overexpressing line OX1 (A) reproducibly exhibits enhanced resistance to OAW, whereas line OX6 does not (B). OX1 and OX6 are independent *DPF*-overexpressing lines. Values are presented as the mean ± standard deviation of biological replicates (n = 4–6) in one representative experiment. Asterisks indicate significant differences between the indicated values (Welch's t-test; * $p < 0.05$ and *** $p < 0.001$).

Supplementary Table S1. List of primers used in this study.

Gene	Primer 1	Primer 2
DPF	CGTGCAAACCTAACATTACA	GGCACCTCCCTTTTTCTTCTT
CPS4	TGACGAGGCTGGGCATATC	TCTGGAGTCCAGTTCCTGAAA
CPS2	CGTGAAGCGTGGAAGCAGT	GCGTAGTCTGCACTGTTCTT
KSL4	GTATTTCATGGGACAAAATCTCTGG	CCATCCTTGCATTCCCTCTC
KSL7	TCTACTACCAGACCGACGGATTC	GAGTTGAAGTGGCTCGTTGATG
KSL8	ATTAATTCCTACGTCTTGTTGCTTGGC	ATATGCGCGGGTTTTCTTCCTAGTTTTAG
KSL10	GCTGATTGAGAAGTGGGATA	GCTGATCCATTATGCTGCGAT
RUBQ1	GGAGCTGCTGCTGTTCTAGG	TTCAGACACCATCAAACCAGA
